# Incorporation, adaptation and rejection of obstetric practices during the implementation of the “Adequate Childbirth Program” in Brazilian private hospitals: a qualitative study

**DOI:** 10.1186/s12978-024-01772-7

**Published:** 2024-04-17

**Authors:** Débora Cecília Chaves de Oliveira, Maysa Luduvice Gomes, Andreza Rodrigues, Thamires Soares, Lucia Regina de Azevedo Nicida, Jacqueline Alves Torres, Elyne Montenegro Engstrom

**Affiliations:** 1 Faculty of Nursing, State University of Rio de Janeiro (UERJ), Rio de Janeiro, RJ Brazil; 2 Anna Nery School of Nursing, Federal University of Rio de Janeiro (UFRJ), Rio de Janeiro, RJ Brazil; 3 Foundation Oswaldo Cruz (Fiocruz), Rio de Janeiro, RJ Brazil; 4 Institute for Healthcare Improvement, Rio de Janeiro, RJ Brazil; 5 Department of Social Sciences at the National School of Public Health, Foundation Oswaldo Cruz (Fiocruz), Rio de Janeiro, RJ Brazil

**Keywords:** Health evaluation, Hospitals, Private, Healthcare models, Quality improvement, Midwifery, Obstetrics

## Abstract

**Background:**

The “Adequate Childbirth Program” (PPA) is a quality improvement project that aims to reduce the high rates of unnecessary cesarean section in Brazilian private hospitals. This study aimed to analyze labor and childbirth care practices after the first phase of PPA implementation.

**Method:**

This study uses a qualitative approach. Eight hospitals were selected. At each hospital, during the period of 5 (five) days, from July to October 2017, the research team conducted face to face interviews with doctors (*n* = 21) and nurses (*n* = 28), using semi-structured scripts. For the selection of professionals, the Snowball technique was used. The interviews were transcribed, and the data submitted to Thematic Content Analysis, using the MaxQda software.

**Results:**

The three analytical dimensions of the process of change in the care model: (1) Incorporation of care practices: understood as the practices that have been included since PPA implementation; (2) Adaptation of care practices: understood as practices carried out prior to PPA implementation, but which underwent modifications with the implementation of the project; (3) Rejection of care practices: understood as those practices that were abandoned or questioned whether or not they should be carried out by hospital professionals.

**Conclusions:**

After the PPA, changes were made in hospitals and in the way, women were treated. Birth planning, prenatal hospital visits led by experts (for expecting mothers and their families), diet during labor, pharmacological analgesia for vaginal delivery, skin-to-skin contact, and breastfeeding in the first hour of life are all included. To better monitor labor and vaginal birth and to reduce CS without a clinical justification, hospitals adjusted their present practices. Finally, the professionals rejected the Kristeller maneuver since research has demonstrated that using it’s harmful.

## Abstract (plain language summary)

Brazil has high Cesarean Section (CS) rates, with rates far from the ideal recommended by the World Health Organization and a model of care that does not favor women’s autonomy and empowerment. In 2015, a quality improvement project, called “Projeto Parto Adequado” (PPA), was implemented in Brazilian private hospitals to reduce unnecessary cesarean section, in addition to encouraging the process of natural and safe childbirth. One of the components of this project was to reorganize the model of care in hospitals to prepare professionals for humanized and safe care. The data were collected in 8 hospitals with interviews with 49 professionals, approximately two years after the beginning of the project in the hospitals. There were changes in the hospital routine and in the care of women after the project. The professionals incorporated practices such as skin-to-skin contact and breastfeeding; diet during labor; non-invasive care technologies, especially to relieve pain during labor; birth plan; pregnancy courses with guided tours in hospitals (for pregnant women and family); and analgesia for vaginal labor. There was adaptation of existing practices in hospitals to reduce CS that had no clinical indication; better monitoring of labor, favoring vaginal delivery. And finally, the professionals rejected the practice that presses the uterine fundus, for not having shown efficacy in recent studies. We can conclude that the hospitals that participated in this study have made an effort to change their obstetric model. However, specific aspects of each hospital, the organization of the health system in Brazil, and the incentive of the local administration influenced the implementation of these changes by professionals in practice.

## Introduction

With global rates of 21.1% and an increase of 12.1% in fifteen years, the use of cesarean section (CS) as an important resource to save lives transcends clinical aspects. The high rates of CS can be influenced by the different political, economic and governmental contexts of each country [1]. In Brazil, even with several investments in the field of obstetric and neonatal care within the Brazilian Unified Health System (SUS), few programs to improve maternal care have been created or expanded in partnership with the private sector [2], although the latter participates in a complementary manner to SUS [3]. As a result of this distance and of a specific cultural context, in 2016 the rate in the country was of 55.44%, with different proportions for the public sector (41%) and the private sector (83%) [4]. Such rates are far from the ideal recommended by WHO (World Health Organization), which is of 10% to 15% [5, 6].

In this sense, the principles guided by the humanization of care and autonomy of women during the process of childbirth and birth [7] has become a struggle for the women’s social movement in Brazil, which through the Public Ministry filed a complaint of Public Civil Action [8] to the National Supplementary Health Agency (ANS), linked to the Ministry of Health (MH) and responsible for creating rules, controlling and inspecting the health insurance market in the country [9], so that there are effective measures for the reduction of unjustified CS in the private health sector.

In response, in 2015, the “Adequate Childbirth Program” (Projeto Parto Adequado—PPA), was launched with the objective of improving the quality of obstetric and neonatal care in the supplementary service, through institutional, scientific and methodological support to hospitals that wished to implement a reorganization regarding prenatal, delivery and puerperium care [10]. In addition to promoting the reduction of CS with no clinical indications, the project promotes a physiological and safe labor and birth process.

To make the intervention feasible, one of the guiding components included the reorganization of the care model, a challenge that carried out the modification and reinvention of care practices, paying attention to the best evidence in obstetrics. Such reorganization depends not only on the adherence of maternity hospitals to the intervention, but also on the adoption or rejection [11] of the implementation by professionals of women’s health care. This article aims to analyze the incorporation, adaptation and rejection of obstetric practices during the implementation of PPA, identified by the care professionals.

## Methods

Recognizing the importance of the intervention PPA to women and newborns health care in Brazil, the National School of Public Health (ENSP) − Oswaldo Cruz Foundation (Fiocruz) proposed an evaluation entitled “Healthy Birth: a prospective study to assess the implementation and effects of multifaceted intervention to improve the quality of care during childbirth and births in hospitals in Brazil” in order to analyze the implementation and the effects of PPA in a sample of 12 hospitals, using mixed methods of analysis [10].

Integrated with this evaluation, this article develops an exploratory study with a qualitative data approach in an intentional subsample of eight hospitals (Hosp01; Hosp02; Hosp04; Hosp05; Hosp06; Hosp09; Hosp10; Hosp12). Inclusion criteria for this subsample were hospital location according to geographic macro-region (South/Southeast/Midwest and North/Northeast), type of hospital (hospitals owned or not owned by health insurance companies), and hospital performance according to administrative data provided by the PPA coordination board. Four hospitals (Hosp03; Hosp07; Hosp08; Hosp11) were excluded due to similarities in geographic location and type of management [10]. More details on data collection, contextual aspects, and protocols established by the “Healthy Birth” survey can be found in Torres et al. [10].

The training of the interviewers who carried out the data collection was carried out in two ways: in person for 02 (two) coaches and remotely for another 02 (two) coaches. In these trainings, there was a reading of the instrument, presentation of techniques for conducting interviews, field observations, and the importance of records.

Regarding the characterization of the 04 (four) interviewers who carried out the data collection: they were all women with academic training in Nursing, Midwifery, and History. The historians were researchers in public health; the nurse and the Midwife were researchers in women’s health; all had previous experience with data collection for research. These interviewees were drawn to the field mainly because they were already involved in women’s health research or care practice. They were presented as such to the interviewees.

Before entering the field for data collection, pilot tests of the scripts were carried out in a maternity hospital in Rio de Janeiro/Brazil, part of the Adequate Childbirth Project. However, this maternity hospital was excluded from the sample of hospitals in the Nascer Saudável evaluation. This phase made it possible to make adjustments and validate the interview scripts. Furthermore, the research team established a previous contact connection with the management of the institutions to agree on the dates on which the interviewers would be placed in the field for data collecting and any ethical issues that would be released.

The immersion for data collection in the hospitals took place during a period of five days, from July to October 2017, in each hospital, in which the research team conducted interviews with the professionals of direct assistance to women. Key informants from the clinical staff (doctors and nurses) were interviewed; they were indicated by the project leader in the hospital among those who were the most integrated and the least integrated to the PPA.

The selection process started with the managers, who nominated leaders and who later nominated the professionals who had, at the time, more and less participation in the project at the hospital (professionals who participated in the PPA alignment meetings and professionals who did not). Physicians and nurses who worked in labor and delivery care at each institution were considered eligible. This selection is called snowball sampling [12] a type of non-probabilistic sampling that focuses on reference chains and is commonly used in exploratory investigations, was used to select participants. A total of 49 interviewees were selected, being 21 doctors (26% women and 18% men) and 28 nurses (all women). The number of respondents was different for each hospital, as it considered the characteristics of each institution. There was no refusal or withdrawal from participating in the research by the interviewees and no need to repeat interviews.

The interviews were carried out in the hospitals, face to face, in a place that guaranteed the participant’s privacy and did not have a pre-established length of time, which varied according to the engagement in the PPA and the subjective characteristics of the interviewees. The interviews were recorded, transcribed by an independent professional, and reviewed by type of sampling by the research team. These transcripts weren’t given back to the participants for review, feedback, or edits. However, for the order of internal validation of the transcripts, there was a review by the research team. Field notes were taken at the end of all interviews in each hospital.

The interviews were guided by a semi-structured script, which included the following axes: decision and participation process; strategies; care practice; and results. This script was developed for Healthy Birth research [10]. With a focus on the care practice axis, the following aspects were explored: the change in the work routine and the way of assisting women in labor, after the beginning of the PPA implementation.

The term ‘saturation’ of qualitative data is incorporated when there are repetitions about the object studied, and it can then interrupt data collection [13]. This term brought ambivalence to researchers, some revealed the feeling of practicality, and others questioned it [14]. It is worth noting that qualitative research, unlike quantitative research, is not based on how many individuals should be heard, but on the intensity of the phenomenon and scope to which the actors are linked to the researched object. This reinforces the importance of refinement in the selection of respondents [15, 16]. Therefore, it is believed that this thesis achieved what the two poles of scholars portray, as it reveals the refinement of the interviewees selected for the interviews, and at the same time, it used the minimum required by many authors, which is the contemplation of at least 20 to 30 interviews for any type of qualitative investigation [17].

Data were submitted to Thematic Content Analysis [18], using the MAXQDA software, version 2020.3 [19]. The interviews were imported into the software, organized and encrypted according to their respective hospital and professional category. The encryption used was identified only in the research dictionary, for greater security with regard to the anonymity of the interviewees. The open categorization was then carried out, generating broad segments. With the refinement of these segments, a list of codes was generated, a step called axial coding. Starting from this list of codes, an inductive association was made to create the categories. The coding tree is available in Table 1.

**Table 1:** Thematic coding tree

**Incorporation of care practices**	Adaptation of the care practices	Rejection of care practices
Information, communication and education	Cesarean	Kristeller Maneuver
Women empowerment information	Elective cesareans over 39 weeks	Practice abolished
Information received by the prenatalist	Clinical justification for the indication of elective cesarean sections	Performed when “necessary”
Information during the maternity tour	Informed Consent Form for cesarean section	Performed, consented to and/or when requested by the woman
Information through the construction of the birth plan	Cesarean at maternal request/desire	
Information received through social media	Indication of cesarean section due to maternal or fetal risk	
Information during labor and delivery	Specific medical teams to perform cesarean/vaginal delivery	
Professional approach to providing information	Absolute indications for cesarean section	
Use of non-pharmacological pain relief methods	There were no changes in the criteria for cesarean section indications	
Squat	Induction of labor	
Therapy bath	Induction of labor, rather than indicating cesarean section	
Swiss ball	Use of Bishop’s index, misoprostol, oxytocin and amniotomy	
Birth stool	Oxytocin	
Walk	Used with discretion	
“Cavalinho” in childbirth	Used by most women	
Professional interaction with the woman during the use of the methods	Used to induce labor	
Skin-to-skin contact and breastfeeding	Used to treat postpartum hemorrhage	
Skin-to-skin contact during cesarean section	There was no change in the indication of oxytocin	
Immediate postpartum breastfeeding	Episiotomy	
Partogram	Practice not performed	
Influence of the partogram on care practice	Perform episiotomy, but selectively	
Partogram update/adaptation needed	Modified episiotomy amount but not because of PPA	
Courses for pregnant women with guided visits to maternity hospitals	There is still resistance to changing practice	
Environment to acquire information		
PPA dissemination environment		
Integration with prenatal care		
Visit and course conducted by the professional nurse		
Number and duration of courses and visits		
Birth plan		
Instrument for providing information		
Instrument that favors previously constructed choices		
Professional approach and respect for the birth plan		
Diet during labor		
Bland diet release		
Insertion of doulas		
Unknown of the concept “doulas”		
The doula hired by the woman (no link with the health institution)		
Presence of companion		
The woman is more comfortable		
Analgesia for vaginal delivery		
Decision on the use of analgesia		
Anesthetist availability		
Indication of analgesia based on cervical dilation		
Creation of a protocol for labor analgesia		
Limitations in the evolution of labor after the use of analgesia for vaginal delivery		

Although there was no feedback from the participants regarding the findings, for the interpretation of the data, the interviewees’ speeches and their respective coding were validated by members of the research group, in which there were also reflexive co-participations on the interpretive procedures of the entire analytical phase [18]. All citations in this article only include the professional position held by the interviewee, to avoid possible identification.

As a methodological guide for qualitative research, it used the consolidated criteria for reporting qualitative research (COREQ) [20].

This research was approved by the Research Ethics Committee, of the National School of Public Health Sérgio Arouca, of the Oswaldo Cruz Foundation (Escola Nacional de Saúde Pública Sérgio Arouca – ENSP/Fiocruz), CAAE opinion: 1.761. 027, on January 16, 2017. The research participants were informed and confirmed their interest in participating by signing the Free and Informed Consent Form.

## Results

**Fig. 1:**
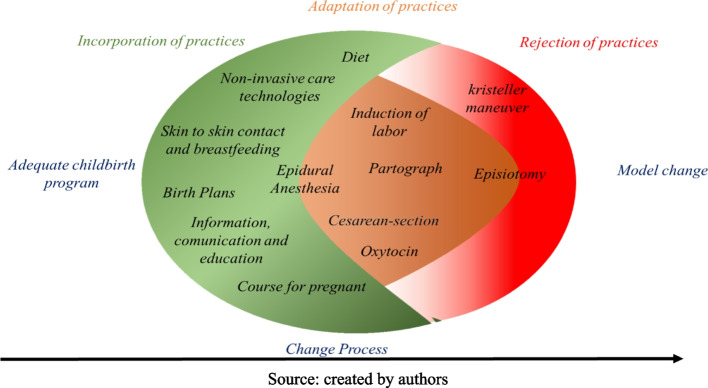
Synthesis of the process of changing the care model in private hospitals in Brazil, after the PPA

The analysis of topics “change in the work routine” and “way of assisting women in labor” after the beginning of the PPA produced three analytical dimensions of the process of change in the care model, described below and synthesized in Fig. 1:

1. Incorporation of care practices: understood as the practices that were included from the implementation of the PPA;
2. Adaptation of the care practices: understood as practices carried out prior to the implementation of the PPA, but which underwent modifications with the implementation of the project;
3. Rejection of care practices: understood as those practices that were abandoned or called into question whether or not they should be carried out by hospital professionals.

### Incorporation of care practices

In this category, we describe the incorporation of care practices by maternity hospitals into the PPA implementation process. Moreover, according to the report of the professionals interviewed, these incorporations signified innovation.

Among the practices incorporated is the provision of: information, communication, and education; birth plan; courses for pregnant women and families with guided visits to maternity hospitals; diet during childbirth; skin-to-skin contact and breastfeeding; non-invasive care technologies; and analgesia for women’s vaginal labor.

Several strategies were used to offer information, communication, and education to women by different professionals and at various moments of their care, which tends to provide subsidies for the strengthening of women’s autonomy and shared decisions, including the reduction of CS for professional convenience.

“When the patient was indicated by her doctor, then it was much more difficult. Today she comes, we already know about the Project, we try to guide her the best possible way. Many come with the idea of having a CS, because their doctor tried to induce it.” (Hosp05_Doctors)

“So much so, that it is not for nothing that we have increased our number of vaginal births. Also because pregnant women are so much better informed. With this Adequate Childbirth Program they already get there with the birth plan, they already give it to the doctor. So the doctor will not arrive at the door and will indicate cesarean because he already sees that she is informed. And the doctor’s posture changes a lot when he sees that the pregnant woman is already informed, that she is empowered, it is different than the one that comes without information. So it has certainly changed.” (Hosp05_Nurses)

Courses for pregnant women with guided visits to the maternity hospitals was another strategy, because it provides subsidies for the recognition by the woman and family of the hospital environment and the health team, in an approach prior to delivery. In addition, the professionals use this space in the course to publicize the maternity hospital’s participation in the PPA, also using it as a marketing strategy to attract customers.

“[…] Our course is not a one-day course. It is a very dynamic course, rich in information. It ends with the guided visit [to the maternity hospital]. It is a monthly course.” (Hosp02_Nurses)

“We also have a monthly pregnancy course, and in this pregnancy course I take the opportunity to publicize the project. So, before finishing the course, I always talk about the PPA, […] what it is and what its purpose is.” (Hosp02_Nurses)

Another strategy carried out by hospitals was the incorporation, encouragement and acceptance of the birth plan made by women. The understanding of the importance of women’s choices during labor and delivery tends to change the focus of attention in obstetrics within the private health sector in Brazil. Currently, the power relationship between medical professionals and women still prevails, which ends up largely disregarding the decisions and planning of women during the gestational and parturient process.

“Obviously, we realize that it is improving. The birth plans are being more respected, in the past they did not exist, and now there is the birth plan for this project. So the least possible intervention is beneficial for the evolution of this labor.” (Hosp 05_Doctors)

“We have a birth plan, which we deliver to patients when they are hospitalized […]. So, when the patient goes into labor and when she is able to answer our questionnaire, we already have this possibility. Every hour, at any time, if we have to do any procedure that escapes our routine a little, the patient will be guided, will be asked at all times. At no time do we take any approach that is not beneficial to the patient, unless there is some risk for the mother and the baby. Then we say: you have to do this procedure, because of this, this and that. But the patient is approached at all times in relation to this.” (Hosp 12 _Nurses)

The diet during labor was described as incorporated by only one professional from one of the eight maternity hospitals, which suggests, considering the context of the country, that there is already an institutionalization of this practice by the other hospitals.

“[…] diet, for example, used to leave every patient in labor on zero diet. Not today. A bland diet is released. So, I can see that this is changing.” (Hosp 02_Doctors)

There was also the introduction of non-invasive care technologies by obstetric nurses, strengthened by the adaptation of the environment and purchase of supplies to be offered during labor and delivery, in addition to being encouraged by medical professionals. These adaptations of the birth environment even modify the logic of control of the bodies for a new model of care, centered on women’s wishes.

“It changed, in the sense that we have more structure to conduct this delivery. So, we have the ball, we have the horse, which they didn’t have before, so when it comes to conducting more effective labor, I think it is favored too.” (Hosp10_Nurses)

“Bath therapy, which previously was almost not talked about, the usual well-being maneuvers in terms of pain.” (Hosp 05_Doctors)

“[…] there are some [women] who just lie down on the stretcher to induce with medication, but then they don’t even lie down, they keep walking, on the ball, on the stool, so it’s different, very different.” (Hosp 02_Nurses)

Another practice adopted was skin-to-skin contact and breastfeeding. Although already well established in Brazilian public institutions, in the private health sector scenario these practices were still not widespread and experienced by women.

“[…] I come from a school where we picked up the baby right away and then, I had it at the time, you know, I aspirated, until the baby had contact with the mother again it would take one hour, at least. So, I completely changed my approach, adapting myself precisely to the rules of PPA. The baby is born, he was born well, let his mother in skin contact. It often goes to the breast before we do the care. So, without any doubt, it completely changed the approach.” (Hosp 02_Doctors)

“So now, this mother / baby contact, breastfeeding is especially important, we are orienting them to breastfeeding, positioning the baby. This is very important. When I came, in the beginning, for pre-delivery, we didn’t have that much about breastfeeding, you know, for an hour.” (Hosp 09_Nurses)

Birth analgesia is between the process of incorporation and adaptation of the practice (Fig. 1), since it had already been incorporated in some maternity hospitals and in others only after the PPA. The availability of anesthesiologists on duty was a major change indicated by professionals to promote the increase of vaginal deliveries in the institutions. Before the PPA, it seemed to be up to the obstetrician to bring or not the anesthesiologist to his team, and consequently to make this practice available to women.

“I think what changed the most was this possibility of analgesia, which is still not possible so soon, but with seven (centimeters of cervical dilation) yes, that already helped a lot. Maybe this analgesia also generates a little more stress, because we know that sometimes you can have fetal bradycardia, so this gives a certain discomfort, but that we keep following.” (Hosp 12_Doctors)

#### Adaptation of care practices

In hospitals where analgesia for vaginal childbirth was already incorporated, the need for adaptations was noted in the professionals’ speeches. For labor analgesia, the most frequently mentioned adaptation related the professionals’ difficulty in valuing the pain threshold and the mother’s desire to indicate labor analgesia, while the cervical dilation score was what substantiated the indication. This strengthens the hypothesis that adaptations to practices may be more difficult to be modified because they are already part of the daily routine and the intrinsic way of doing things of each professional.

“[…] most [professionals] do analgesia only with total dilation, or when it’s very close to giving birth. Obviously, there are some cases that do with seven (centimeters of cervical dilation), but this is the minority of cases. So, when she does analgesia, the patient is already in the room to get a baby. Usually, they put Oxytocin, even if she was not conducting the delivery with Oxytocin before, usually they put it in the room, because when she does analgesia, the contraction is lowered and she has this difficulty to feel because of the block [..].” (Hosp 09_Nurses)

“So, we also have a lot of work, because it is like this: at first it is the obstetrician who decides the analgesia. It is not the woman. It’s not the woman’s pain, it’s the obstetrician. […] This is being changed too.” (Hosp 05_Nurses)

Regarding the use of the partograph, a tool that promotes a follow-up of labor, it has been incorporated more frequently during the PPA. However, at the time of the implementation of the PPA, the recommendation used was not the most recent in the literature. This allows us to reflect on the benefits of the insertion of this practice, but with the need to adapt to the best evidence. This reflection on adaptation is raised by the professionals.

“The use of the partograph for assistance in labor was significantly higher.” (Hosp 04_Doctors)

“[…] we still have a tool that is obligatory, and we must fill in, which is the partograph that I think is a great advance and is one of the tools of PPA, but it is still in the model of the WHO, which I think should be updated […]. Because today is it like this: every two hours you have to play it. It is a guidance. There are already some guidelines that guide it for every four hours, but less than four hours there is no need. I joke with the nurses: the finger doesn’t have an eye.” (Hosp 05_Doctors)

The perspective of CS indications was also modified after the PPA. It included mechanisms such as indicating elective CS to be performed only above 39 weeks of gestational age, requiring professional justification for performing elective CS below 39 weeks, and the adoption of a consent form for women to be informed and authorize the surgery.

“[…] there is a whole training for this. And it’s barred since the call, when the doctor calls to schedule [the CS], always ask: elective CS, how many weeks? But thirty-seven weeks, why? Why are you asking for a vacancy (to perform the surgery)?” (Hosp 01_Doctors)

“There has been a change. There is a CS form that the patient has to sign, or the husband.” (Hosp 02_Nurses)

In order to reduce the number of CS and promote vaginal birth there were consequently more cases of induction of labor. Therefore, it has been necessary, for example, to acquire specific drugs for induction, such as Misoprostol.

“[…] I can mention the increase in the rate of labor induction for patients, instead of CS.” (Hosp 04_Doctors)

“A patient who arrived with an unfavorable Bishop score […] outside of labor, sometimes ended up in a CS, because we didn’t have this device [Misoprostol]. Now, having it, improved.” (Hosp 09_Doctors)

In this sense, the routinely use of synthetic Oxytocin also changed. Professionals of most hospitals reported a change in culture, for use only in cases of induction of labor, in the postpartum, and for cases of postpartum hemorrhage. However, two professionals of Mat06, of all the interviewees, expressed that this practice has not changed after the PPA in the institution where they work.

“A lot of changes. Today Oxytocin is used is for an induction, if it’s a postpartum, or something. […] Use Oxytocin to accelerate, there’s no more of that.” (Hosp 05_Nurses)

“But then, when i was hired by the hospital, Oxytocin use was routine. Does not do a dynamic and it already starts with Oxytocin. I didn’t have the right protocols to follow, you know? But you realize that this is already changing.” (Hosp 05_Nurses)

“Oxytocin, […] I believe it hasn’t really changed, because I think that those who used it continue to use it.” (Hosp 06_Doctors)

The practice of episiotomy is still performed in all hospitals. However, according to the reports, there has been a decrease in its use, with most professionals, according to them, using it in a “selective” way. Even if slowly, professionals began to question the use of episiotomy, since there is no scientific evidence of its effectiveness. However, it is still far from a unanimous acceptance.

“Episiotomy I was already using, selective episiotomy. When I was doing residency, we did seventy percent episiotomy. Now, today, we do twenty to thirty percent of episiotomy. So there is already a selection.” (Hosp 06_Doctors)

“Episiotomy, it is always at the discretion and when we see that there is a need, we ask the patient: do you authorize me to do the episiotomy? This is very controversial, in fact, because currently there are people who say they don’t really need it, anyway, but when it is done, it is done with discretion and asking: do you authorize?” (Hosp 09_Doctors)

“I no longer see a doctor doing an episiotomy.” (Hosp 04_Nurses)

“Performing episiotomy, is a maneuver no longer used by our entire team.” (Hosp 04_Doctors)

#### Rejection of care practices

Most professionals have reported abandoning the practice of Kristeller. However, for the few who still mention the use of this practice, it has remnants of a need to take possession of the female body. This category demonstrates how difficult it is to let go of obsolete practices, which still conform a violent obstetric care, less woman-centered and exercised without scientific base. Therefore, we understand that the (de)construction of practices seems to be daily and continuous in hospitals.

“Kristeller has been abolished. We still used it in some situations, before the project and today it has been abolished. I never saw it again […]. We started talking, like this, slowly, asking not to do it, in a nice way.” (Hosp 06_Nurses)

“Kristteller, I think I only saw it once. Because help was needed. But he (the doctor) talked to the woman.” (Hosp 05_Nurses)

Below (Fig. 1) we present a synthesis of the results with the analytical categories of incorporation, adaptation and rejection of practices for the change of the obstetric model in private hospitals after the implementation of PPA.

## Discussion

The model of obstetric care in force in Brazil is still predominantly marked by obsolete interventionist care practices that, for the most part, do not value women’s wishes and expectations. An epidemiological survey conducted in the Brazilian territory, entitled “Born in Brazil”, in 2011/2012, found an excess of unnecessary obstetric practices during labor and delivery, with only 5% of women at usual risk without any intervention during the birth process [21]. This finding reinforces the need to change the obstetric care model in force in the country, especially in the private sector. Thus, the Adequate Childbirth Program encourages and promotes the incorporation, adaptation, and rejection of practices based on the best evidence in private hospitals in Brazil.

Thus, the intrinsic innovation of PPA is in the promotion of this new model of care, which corresponds to the transformation of the scenario, using technologies, processes, ways of caring and managing [22] in obstetric health institutions. Innovations can be carried out incrementally to what already existed in the context, or they can be implemented radically, excluding what existed before [23]. In the case of hospitals inserted in the PPA, we note that the change of model, still in a continuous process, occurs incrementally, as shown in Fig. 1.

In the context of each hospital inserted in the PPA study presented in this work, there was the incorporation of practices that were not yet institutionalized, and one of them was the promotion of information, communication, and education in health [24]. WHO’s guidelines recommends that there is clear communication between health professionals and parturient, thus ensuring the recognition of their rights regarding the entire birth process [25]. However, a study conducted in Brazil in 2020 revealed that women still remain uninformed about the benefits and harms of obstetric care practices, which ends up hindering their autonomy at the time of labor and the claims of their rights [26].

Corroborating the WHO guidelines [25] and keeping in mind that this is an ongoing process, the initiative of hospitals to offer health education, such as courses for pregnant women, maternity tours, and stimulus to the construction of the birth plan, exemplifies strategies to increase information, knowledge of women and better prepare them for the birthing process, and this includes inhibiting medically unjustified CS.

The incorporation of the use of practices with Non-Invasive Technologies of Care [27] goes through a change in professional behavior, in which the care relationship is modified and becomes shared, centered on the woman and the family. Such incorporation modifies the whole perspective of the current model, because in this new model, the skills and competences of the professional who assists the woman enhance her femininity in the scenario of labor and birth. In this context, in a sensitive and individual way, the development of non-invasive technologies of care involves relational attitudes, professional knowledge in obstetrics and the offer of objects and procedures such as the ball, the horse, therapeutic bath, music therapy, among others [27, 28].

This corroborates the results of this study, which shows that professionals perceive the woman’s satisfaction when using these technologies. This path of change must be built daily in women’s care settings, because many are still unaware of the importance of freedom of position during labor [29], besides the possibility of giving birth in vertical positions, as well as the possibility of feeding during labor.

Proper monitoring of labor is essential for safe maternal and neonatal outcomes. The use of partographs as a labor monitoring tool is a strategy encouraged by health agencies worldwide. However, in recent years, the partograph’s warning and action lines have been questioned as to their effectiveness because not all women progress 1 cm/hour during the first stage of labor. Some women progress more slowly. Recognizing the limitations of this tool and proposing the creation of an assessment that can better individualize women, the WHO recommendation is to continue to use the partograph’s warning and action lines and its other aspects of labor assessment, but for women with suspected slower labor progression, it should be ensured that their physical and psychological needs are being met, to avoid misdiagnosis of cephalo-pelvic disproportion [25].

In a review on the use of partography during work, the lack of training of professionals to use the instrument, the lack of time to execute it properly, as well as the lack of institutional protocols to guide the conduct on the results of the partography were highlighted [30]. Given this and the reports of professionals, clinical updates will be necessary for the use of partography in hospitals inserted in the PPA.

Another recommended practice for pain relief, in healthy women who request its use, is pharmacological labor analgesia [25]. In Brazil, this right is guaranteed by the ordinances of the Ministry of Health NR2815/1998 and NR572/2000 [31, 32]. Regional analgesia by continuous or combined epidural technique with reduced doses is considered the gold standard [32], mainly because it provides effective pain relief, without removing the woman’s participation during labor, it avoids maternal hyperventilation and increases maternal satisfaction [33].

Studies on the association of pharmacological analgesia for vaginal delivery with unfavorable neonatal outcomes showed that it can increase by about 3 times the chance of Apgar score of the newborn in the first minute to less than 7. However, in the Apgar score of the fifth minute there was no such association, which may be related to the preparation of the team in relation to resuscitation maneuvers [32, 34]. Thus, the concern of the professionals interviewed is valid, when they understand that there is maternal satisfaction due to pain relief; however, there is also the need to prepare the team for neonatal care, when necessary.

Globally, CS rates tend to be higher in countries with more doctors in the territory; and in women living in large urban centers, with higher levels of economic development, with higher education and with lower fertility rates. Comparing wealthy women, especially those who give birth in private maternity hospitals, one notices higher rates of CS compared to poorer women [1]. In a local study, women more likely to have CS are those with higher levels of education, who live in places with better living conditions, who attend prenatal care, who are over 20 years old, and who have partners. In Brazil, this happens not only because of issues related to access to health services, but also because of contextual aspects of the health system organization, the obstetric care model, and sociocultural factors [4].

In this sense, even with the adjustment to the Brazilian population of the CS rate to about 25–30%, through the WHO C-Model [32], the development of various strategies in the field of obstetrics is still necessary to reduce the high rates of CS [35]. Understanding this, the hospitals inserted in the PPA started with good strategies to decrease CS rates that can serve as an example for other institutions.

Skin-to-skin contact regulates and maintains the baby’s body temperature and cardiorespiratory stability soon after birth. This practice provides opportunities for breastfeeding in the first postpartum hour and tends to strengthen the emotional bond between mother and baby, and short episodes of crying and signs of stress in the newborn [36–38]. Moreover, skin-to-skin contact also stimulates the newborn to spontaneously seek the breast and start early breastfeeding.

This last practice is encouraged by the World Health Organization and the United Nations Children’s Fund (UNICEF) when it establishes the Ten Steps for Successful Breastfeeding as the basis for the Baby-Friendly Hospital Initiative [39], by promoting maternal and neonatal benefits. Among the immediate benefits are the stimulation of maternal endogenous oxytocin release, which favors uterine contraction, reducing postpartum bleeding, and consequently, reducing maternal postpartum hemorrhage [36, 38]. The incorporation of the practices of skin-to-skin contact and breastfeeding by the professionals of hospitals inserted in the PPA, also reinforces the change in the care model, centering care on the woman and the newborn.

With regard to episiotomy, the literature recommends its selective use in women with spontaneous vaginal delivery. However, as there is no evidence to support the effectiveness of its use, even if selectively, the most recent WHO manual preferred to emphasize that its routinely use is not recommended. Moreover, it reinforces that in cases where the procedure must be performed, local anesthesia and the woman’s consent are essential [25]. In Brazil, the use of episiotomy is still extremely high, varying from institution to institution, but there is a higher frequency of this practice especially in primiparous women and more frequently in the private sector, even if the woman is considered of usual risk [21].

WHO’s recommendations [25] highlight the evidence that women are afraid of interventions such as episiotomy and that when there is a need for this practice to be “indicated”, such women would like to have more information about the procedure, in addition to it being performed by a competent professional. Furthermore, most women do not have the necessary information about why the procedure should be performed and what care should be taken afterwards, and they rarely have their permission requested [25]. The speech of hospital professionals included in the PPA reinforces WHO’s proposal, but still in a transition phase between the adequacy of the practice and its rejection. This path of change is arduous and difficult to achieve due to the fact that this practice in considered as normal in Brazilian institutions, since the idea that episiotomy facilitates childbirth still exists.

The use of the Kristeller maneuver at the time of fetal expulsion is contrary to the best evidence, suggesting that there is little or no difference in the time of fetal expulsion when pressure is applied or not to the uterine fundus. In addition, low certainty evidence suggests that women who receive the Kristeller maneuver may experience more pain after birth, and that birth trauma, including fractures and bruising, may occur. Furthermore, concerns regarding the practice of uterine pressure are due to the possibility of serious harm such as uterine and other organ rupture, and maternal and perinatal death. The use of such practice without seeking the woman’s consent may be considered an abuse of human rights [25].

Between 2011 and 2017, there was a considerable increase in the proportion of women who had access to technologies for labor and delivery (presence of a companion, attendance by an obstetric nurse, use of partogram, use of non-pharmacological methods, ambulation during labor and delivery, feeding and position for delivery) and decline in practices such as peripheral venous catheter use, episiotomy, and Kristeller maneuver. The private sector also observed declining cesarean rates and increasing gestational age at birth. The study’s findings, which compared two improvement programs in the Brazilian obstetric industry, demonstrate that well-designed public policies can change how care is provided during labor and delivery, helping to reduce adverse outcomes for mothers and newborns [40].

Initiatives to increase the information and participation of women and to modify hospital routines can be strategies to reduce cesarean sections, used mainly in high-income countries, such as changing the form of reimbursement to health institutions and organizations; and creating or strengthening mechanisms for legislatures and politicians to bar medically unjustified elective cesareans and stimulus to the construction of the birth plan [40–44].

The lack of proportion of participants in “more integrated” and “less integrated” to the PPA can be considered a methodological limitation of the study. In addition, not known whether such changes presented in the results also happened due to other political, legislative, and women’s movement interventions that may have occurred concomitantly in the country or health institutions. Future studies that reflect the implementation of the PPA from other perspectives, such as the voices of maternity heads and women, are necessary. Many of these articles are already in the process of being published by the research team.

## Conclusion

After the PPA, there were adjustments made to the hospital’s routine and the care given to women. Skin-to-skin contact, breastfeeding, food during labor, non-invasive care technology, birth plans, prenatal with guided hospital visits (for expectant mothers and their families), and analgesia for vaginal delivery included by professionals. Hospitals modified their current procedures to decrease CS without a clinical reason and to better monitor labor and vaginal birth. And lastly, because recent research has shown that the practice of applying the Kristeller maneuver is ineffective, the professionals rejected it. We can draw the conclusion that the hospitals included in this study have tried to alter their obstetric model.

It is expected that these practices, which are constantly changing, will produce positive impacts on the obstetric care model and, consequently, on the safety and satisfaction of women in the labor and birth process. The contextual aspects of each hospital, the organization of the health system, and the management stimulus has influenced the process of change through the PPA implementation.

Therefore, the sustainability of such change transitions in a long-term culture modification, professional training, continuous monitoring and evaluation of clinical practice, in addition to the influence of hospital management in the face of innovation. It is concluded that the professionals interviewed reported a reorganization of the ways to know and care for obstetrics after the implementation of the PPA.

## Data Availability

The datasets used during the current study are available upon request at: Leal, Maria do Carmo (Coord.), 2023, "Nascer Saudável: estudo prospectivo de avaliação da implantação e dos efeitos de intervenção multifacetada para melhoria da qualidade da atenção ao parto e nascimento", https://doi.org/10.35078/C1PSMZ, Arca Dados, V2, UNF:6:LQBoC/LmVRpwUe/UvaYCfQ== [fileUNF]

## Abbreviations

ANS: National Supplementary Health Agency
CS: Cesarean Section
ENSP: National School of Public Health
IHI: Institute for Healthcare Improvement
MH: Ministry of Health
PPA: Adequate Childbirth Program (“Projeto Parto Adequado”)
SUS: Unified Health System
WHO: World Health Organization
